# Long term treatment with abatacept or tocilizumab does not increase Epstein-Barr virus load in patients with rheumatoid arthritis - A three years retrospective study

**DOI:** 10.1371/journal.pone.0171623

**Published:** 2017-02-15

**Authors:** Nathalie Balandraud, Gaetan Texier, Emmanuel Massy, Olivier Muis-Pistor, Marielle Martin, Isabelle Auger, Marie-Caroline Guzian, Sandrine Guis, Thao Pham, Jean Roudier

**Affiliations:** 1 Rheumatology 1 or 2, IML, AP-HM, 270 Boulevard de Sainte Marguerite, Marseilles, France; 2 INSERM UMRs 1097, Aix-Marseille University, 163 Avenue de Luminy, Marseilles, France; 3 CESPA, Centre d'épidémiologie et de santé publique des armées, Marseilles, France; 4 Aix-Marseille University, INSERM, IRD, SESSTIM, Sciences Economiques & Sociales de la Santé & Traitement de l’Information Médicale, Marseilles, France; University of North Carolina at Chapel Hill, UNITED STATES

## Abstract

**Background:**

Epstein-Barr Virus (EBV) is a widely disseminated lymphotropic herpes virus implicated in benign and malignant disorders. In transplant patients, immunosuppressive drugs (cyclosporine) diminish control of EBV replication, potentially leading to lymphoproliferative disorders (LPD). Rheumatoid arthritis (RA) patients have impaired control of EBV infection and have EBV load ten times higher than controls. As post transplant patients, patients with RA have increased risk of developing lymphomas. Immunosuppressive drugs used to treat RA (conventional disease modifying drugs cDMARDs or biologics bDMARDs) could enhance the risk of developing LPD in RA patients. We have previously shown that long term treatment with Methotrexate and/or TNF alpha antagonists does not increase EBV load in RA. Our objective was to monitor the Epstein-Barr Virus load in RA patients treated with Abatacept (CTLA4 Ig), a T cell coactivation inhibitor, and Tocilizumab, an anti IL6 receptor antibody.

**Methods:**

EBV load in the peripheral blood mononuclear cells (PBMCs) of 55 patients under Abatacept (in 34% associated with Methotrexate) and 35 patients under Tocilizumab (in 37% associated with Methotrexate) was monitored for durations ranging from 6 months to 3 years by real time PCR. The influences of treatment duration and disease activity score 28 (DAS28) index on EBV load were analyzed.

**Results:**

Abatacept did not significantly modify EBV load over time. Tocilizumab significantly diminished EBV load over time. No patient (of 90) developed EBV associated lymphoma.

**Conclusion:**

Long term treatment with Abatacept or Tocilizumab does not increase EBV load in the PBMNCs of patients with RA.

## Introduction

Epstein-Barr Virus (EBV) is a widely disseminated lymphotropic herpes virus implicated in benign and malignant disorders. EBV infects B lymphocytes and epithelial cells. Once EBV's initial lytic infection is brought under control, EBV persists in the individual's B cells for the rest of the individual's life [1].

Rheumatoid arthritis (RA) is one of the most common autoimmune diseases with a 0.5% world-wide prevalence. Patients with rheumatoid arthritis have impaired control of EBV infection. Indeed, they have high-titre antibodies to EBV antigens [2]. Their peripheral blood T lymphocytes are less efficient at controlling the outgrowth of EBV-infected B cells [3]. RA patients have more EBV-infected B cells than normal controls [4]. Disease activity is associated with lower T cells responses to the EBV replication protein gp110 [5]. Impaired control of EBV infection leads to ten fold systemic EBV overload in RA patients, very much like what is observed in healthy organ transplant recipients [6].

Both RA patients and solid organ transplant recipients are at increased risk of developing lymphoma [7]. In solid organ transplant recipients under immunosuppressants, emergence of lymphoma can be predicted by monitoring EBV load in peripheral blood mononuclear cells (PBMCs) [8]. Post-transplant lymphoproliferative disorder (PTLD) is a polyclonal EBV positive B lymphocyte proliferation which can evolve into EBV positive B cell lymphoma [9]. EBV load above 500 copies per 500 ng PBMC DNA is considered a limit above which patients may develop PTLD. Increase in EBV load may be more predictive of PTLD than load itself, making EBV load monitoring over time a good method to detect early PTLD [10].

RA treatments have evolved in the last twenty years with the widespread usage of methotrexate, then the development of TNF alpha inhibitors. Both were suspected to increase the risk of developing lymphoma. We monitored EBV load in RA patients under these treatments and found that long term usage of methotrexate or TNF inhibitors does not increase EBV load and is associated with reduced risk to develop lymphoma [11].

New treatments acting on new targets, such as Abatacept (CTLA4 Ig), a T cell coactivation inhibitor [12–15] and Tocilizumab, an anti interleukine 6 receptor antibody [16–20] are now commonly used.

EBV load monitoring under Abatacept (CTLA4 Ig) is especially relevant because abatacept inhibits T cell activation, as immunosuppressive drugs (cyclosporine) used in organ transplant do. Recently, Belatacept, a mutated abatacept molecule with higher immunosuppressive potency used in renal transplant was found to increase EBV replication and the risk for central nervous system B lymphoma [21,22]. In RA patients, T cell responses to EBV are impaired and could be worsened by Abatacept, leading to lymphoproliferative disease.

Tocilizumab is a monoclonal antibody that competitively inhibits the binding of IL-6 to its receptor. IL6 is involved in viral immunosurveillance by stimulating hematopoietic cells. Mourgues et al. monitored EBV load in 20 patients with RA treated by tocilizumab and found no increase [23]. Fujieda et al. followed Epstein-Barr virus load for three years in peripheral blood lymphocytes patients with juvenile idiopathic arthritis treated with methotrexate and tocilizumab and found it to decrease [24].

Finally, EBV load monitoring after immunosuppressive therapy is crucial in transplant patients because rapid increase of EBV load is associated with high risk of lymphoma. Thus, monitoring EBV load during long term RA treatment should predict the risk of developing immunosuppression EBV positive B cell lymphoma. Here, we monitored EBV load in 55 RA patients under Abatacept (in 34%, associated with Methotrexate therapy) and 35 patients under Tocilizumab (in 37%, associated with Methotrexate therapy) for durations ranging from 6 months to 3 years.

## Materials and methods

### Patients (Table 1)

**Table 1:** Patients characteristics.

	Patient	Woman number	Age	ACPA^+^	RF^++^	SE^+++^	Disease duration	MTX	DAS 28 at	Number of
	Number	(%)	(years)	(%)	(%)	(%)	(years)	(%)	begining	Biologics^++++^
**Abatacept**	55	47 (85)	57+/-12	37 (67)	44 (80)	33/52 (65)	15+/-9	19 (34)	4.58	2.2
**Tocilizumab**	35	33 (94)	51+/-14	28 (80)	28 (80)	21/30 (70)	12+/-11	13 (37)	4.77	2.5
**Tocilizumab after Abatacept**^**+++++**^	12	11 (91)	60+/-14	6 (67)	9 (75)	7/12 (59)	16+/-12	3 (25)	4.95	3.3
**All**	90	80 (88)	55+/-13	65 (72)	72 (80)	54/82 (66)	13+/-10	32 (35)	4.65	2.3

Values are mean ± SD. SE = Shared Epitope.

^**+**^ Anti citrullinated peptide antibodies

^**++**^ Rheumatoid Factor

^**+++**^ = number of Shared Epitope positive/nb tested

^**++++**^ Number of biologics used before the beginning of the study

^**+++++**^ Tocilizumab after abatacept is a subgroup of 12 patients among the 35 patients that have been treated with tocilizumab after abatacept.

Ninety patients with RA (satisfying the 1987 ACR criteria [25] since the 2010 ACR criteria were not available when we started this study) from the Rheumatology department at Hôpital Sainte Marguerite, Marseille, were followed for durations ranging from 6 months to 3 years. Recruitment period was from September 2009 to April 2015.

Fifty five patients received IV Abatacept (500 to 750 mg/ 4 weeks). In 19 (34%), Abatacept was associated with methotrexate (7.5–15 mg/week). Thirty five patients received IV tocilizumab (8mg/kg/ 4 weeks). In 13 (37%), tocilizumab was associated with methotrexate (7.5–15 mg/week). Twelve patients first included in the abatacept group, were switched to the tocilizumab group during the study, and were analyzed in both groups. Baseline characteristics of patients are presented in **Table 1**. Viral load was measured before the first injection of abatacept and tocilizumab. In patients who were treated with a second line of biologics, a treatment free period (5 half lifes of previous drug) was implemented before switching to the new biologic.

Peripheral blood samples were taken every 6 months for EBV DNA assay. Clinical data and DAS 28 score [26] were also analyzed.

### DNA preparation

As previously described [6,11], Human genomic DNA was isolated from 10 ml of heparinized blood. Mononuclear cells were isolated by isopycnic centrifugation through Ficoll-Histopaque (Sigma, St Louis, USA) and processed through Qiagen Genomic-tips 100/G (Qiagen, Courtaboeuf, France) according to the Qiagen genomic DNA handbook. DNA was resuspended in 10 mM Tris, pH 8 and was quantified by real time PCR with a LightCycler (Roche, Mannheim, Germany) as previously described [6].

### Quantification of EBV copy number

As previously described [6,11], 500 ng DNA from peripheral blood lymphocytes was used for EBV DNA assay by quantitative real time PCR (QRTP). Briefly, a 214bp segment of the highly conserved long internal repeat region 1 (IR-1) of EBV was amplified by QRTP using hybridisation probes: (Tib-Molbiol, Berlin, Germany) and a Lightcycler^@^. The Raji cell line was used as an external EBV standard. Each sample was tested in duplicate and assayed twice. For final results, EBV copy numbers in test samples were calculated for 500 ng DNA.

### Statistical analysis

We estimated the effect of treatment (Tocilizumab & Abatacept) on EBV load and on the Disease Activity Score in 28 joints (DAS28) by a population-averaged model: the generalized estimating equations (GEE) model [27]. GEE are typically used to describe changes in the population mean by taking into account the dependency of the observations provided by the same patient. We used, as correlation structure, a first-order autoregressive structure (assuming a steady decay in correlation with increasing time or distance between observation). Analysis was realized with R software 3.3.0 [28] and we fit the GEE model using the geeglm function included in the R package geepack [29].

### Ethics

Sample collection and analysis (2009-A00247-50) for 80 patients was approved by the Ethics commitee « CCP Sud Méditerrananée 1 » in June 2009. In 2012, EBV monitoring became routine in our division, so 10 supplementary patients could be included. Sample collection and analysis (DC-2008-327) was approved by the « Cellule Bioéthique, Direction Générale pour la Recherche et l’Innovation, Ministère de l’Enseignement Supérieur et de la Recherche). For patients, serum samples were rendered anonymous before analysis. All participants gave written informed consent.

## Results

EBV load was monitored in 90 RA patients treated with Abatacept +/- methotrexate (55 patients) or Tocilizumab +/- Methotrexate (35 patients) for durations ranging from 6 months to 3 years. Our population was typically a population of long standing RA (mean disease duration 13 years). This population fullfiled the 1987 ACR / EULAR criteria and not the 2010 ACR / EULAR criteria, explaining the small proportion of patients with anti citrullinated protein antibodies (67%) compared to newly diagnosed RA (more than 80% expected). RF is present in 80% as usually described. Most of the patients were treated with a second line biologic. All prior biologics were TNF alpha inhibitiors. No patient had received rituximab or anakinra.

### Abatacept does not increase EBV load

**Fig 1:**
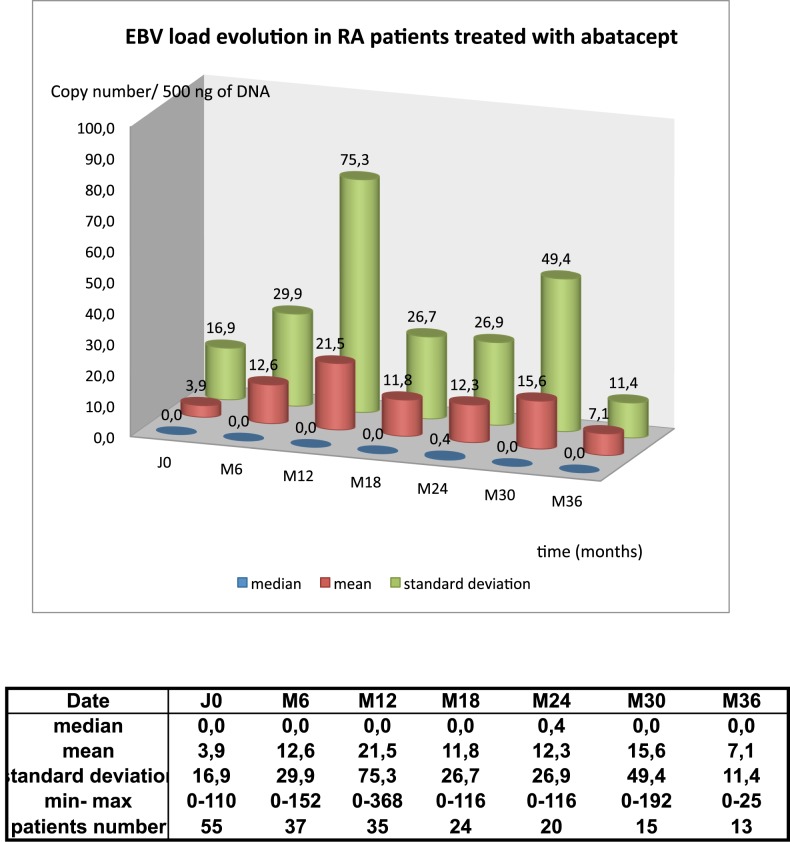
Mean EBV load in RA patients treated with Abatacept. Epstein Barr virus copy number per 500ng PBMC DNA was assayed in 55 patients under abatacept. For all patients, EBV load, median and standard deviation are given every 6 months from the beginning till 36 months. Four patients accepted to be followed for one more year.

EBV load was monitored in 55 patients under abatacept. At baseline, EBV was detectable in 11 of 55 patients. EBV loads ranged from 1 to 110 copies/500ng DNA, mean load was 3,85 copies/500ng DNA. Over time, EBV load remained stable in 50 patients and increased slightly over 100 copies to decrease later under 50 copies in 5 patients. At the end of the study, EBV loads ranged from 0 to 36 copies/500ngDNA, mean load was 8,7 copies/500ng DNA and median load was 2,74 copies /500ng DNA. **(Fig 1 and S1 Table).**

**Fig 2:**
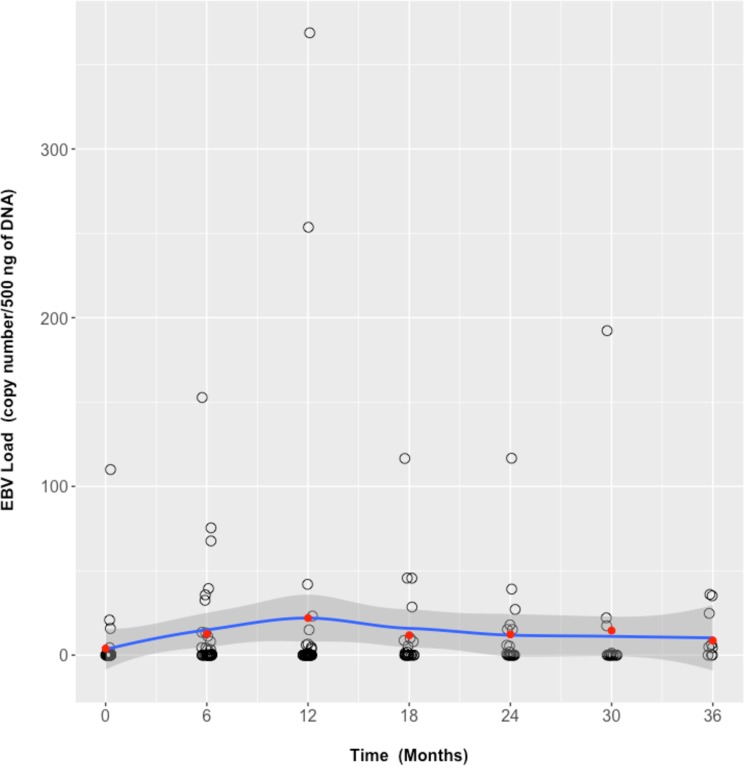
EBV load evolution under abatacept treatment. Each black circle represents a patient’s EBV load. Red dots indicate mean EBV load every 6 months. Mean EBV load evolution is drawn as a blue line +/- 1 standard deviation (dark grey surface) displaying the LOESS (LOcally wEighted Scatter-plot Smoother) regression.

We estimated the effect of treatment on EBV load by GEE population-averaged model. Abatacept was not significantly (p = 0.715) associated with increased EBV load over time **(Fig 2).**

### Tocilizumab decreases EBV load

**Fig 3:**
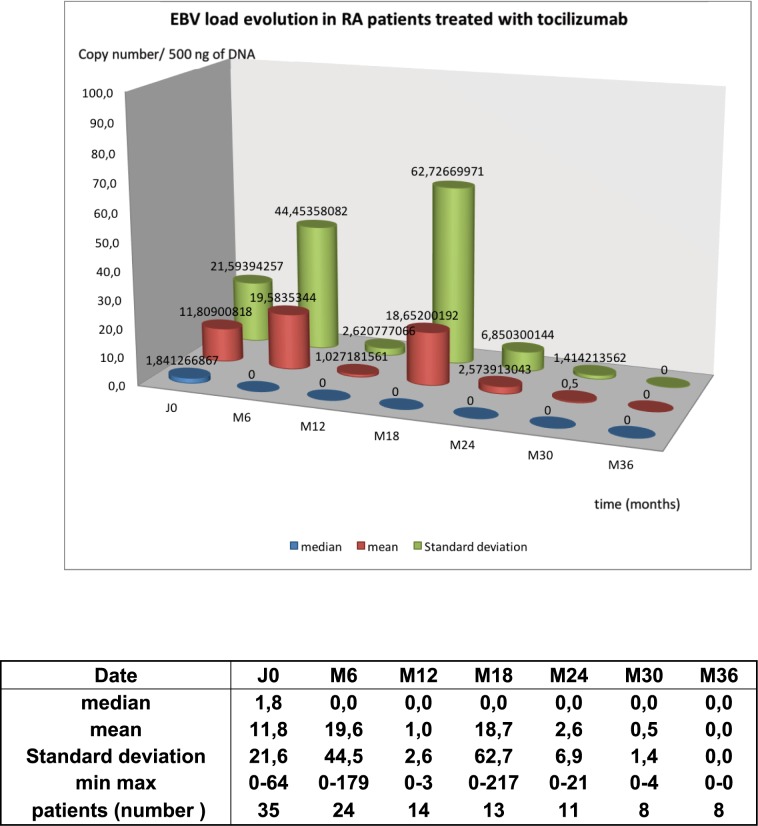
Mean EBV load in RA patients treated with Tocilizumab. Epstein Barr virus copy number per 500ng PBMC DNA was assayed in 35 patients under tocilizumab. For every patient, EBV load, median and standard deviation are given every 6 months from the start till 36 months. Four patients accepted to be followed for one more year.

EBV load was monitored in 35 patients under tocilizumab. At baseline, 24/35 patients had detectable EBV (70%). EBV loads ranged from 0 to 64 copies/500ng DNA, mean load was 12 copies/500ng DNA. Under treatment, EBV load remained low or decreased in 19 patients. In one patient, EBV load increased from 38 to 238 copies/500ng DNA at 6 months. This patient could not be followed any longer because he moved from our center. At Month 24, 11 patients were still followed and only two had detectable levels of EBV DNA. At month 36, none of the 8 patients that were still followed had detectable EBV DNA **(Fig 3 and S2 Table).** At month 48, no patient (on 5) had any detectable EBV DNA (data not shown).

**Fig 4:**
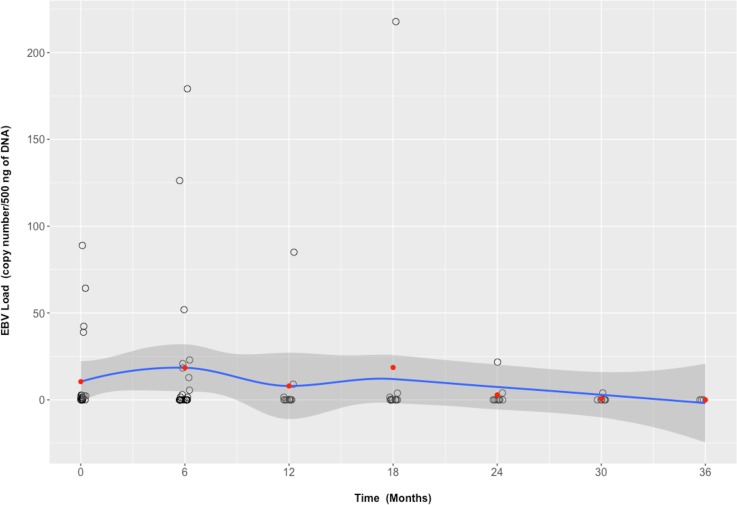
EBV load evolution under tocilizumab treatment. Each black circle indicates EBV load in one patient. Red dots indicate mean EBV load. Mean EBV load evolution is drawn as a blue line +/- 1 standard deviation (dark grey surface) indicating the LOESS (LOcally wEighted Scatter-plot Smoother) regression.

We estimated the effect of tocilizumab on EBV load by a population-averaged model. We showed that under tocilizumab, EBV load significantly diminished (p = 0.021) **(Fig 4).**

### Association with methotrexate therapy

Under abatacept and tocilizumab respectively, 19/55 (34%) and 13/35 (37%) patients were treated with methotrexate. We compared EBV load evolution in patients treated with or without methotrexate and could not see any significant difference between the two subgroups (data not shown), although the number of patients per subgroups was small.

### Twelve patients received tocilizumab after abatacept

They were re-analyzed as an independant group. In these patients, after abatacept therapy and before tocilizumab treatment, EBV loads ranged from 0 to 64 copies, mean load was 19,7 copies/500ng DNA. After tocilizumab treatment, EBV was not detectable excepted in one patient who had 89 copies/500ng DNA. No difference could be seen between patients that first received abatacept or tocilizumab (Data not shown).

### Absence of association between DAS28 and EBV load

Whenever possible, disease activity was evaluated by calculating the DAS28 index. This resulted in a total 529 blood samples for which we had both EBV load and DAS28. Both treatments diminished the DAS 28 over time.

No association (p = 0.110 for abatacept and p = 0.149 for tocilizumab) was found between EBV load and DAS28 by Generalized Estimation Equations. (See S1 Fig, S2 Fig).

## Discussion

### Rheumatoid arthritis, lymphoma, EBV

RA patients are at increased risk of developing lymphoma [30–33].

In RA patients, impaired control of EBV infection results in a 10 fold increased EBV load in peripheral blood mononuclear cells [6], as observed in healthy transplant recipients under immunosuppressants [34,35].

The EBV status of lymphomas in RA patients is still controversial. A study of 343 lymphomas which occurred between 1964 and 1995 in RA patients from Sweden before TNF blockers were used demonstrated that, although most lymphomas were B cell derived, only 12% contained the EBV genome [31]. A study in France found increased incidence of EBV positive Hodgkin’s lymphomas (most of them EBV positive) under methotrexate, but no increased incidence of diffuse B cell lymphomas [36]. This was consistent with our observation that methotrexate tends to decrease EBV load in RA patients [6]. This is very different from lymphomas in transplant recipients which are usually EBV positive.

In transplant recipients, B cell lymphomas usually contain the EBV genome. They are preceded by polyclonal expansion of EBV positive B cells, hence the increase of EBV load detectable in peripheral blood lymphocytes before the emergence of lymphoma. Many kits to quantify EBV load are available today, some quantify EBV in whole blood, others in PBMCs, making comparison very difficult. To this day, no consensus on the way to quantify EBV exists. Still, most authors acknowledge that a load higher than 500 EBV copies per 500ng PBMC DNA and still rising is a good predictor of lymphoma onset [10,37].

To monitor EBV load in RA patients under immunosuppressants, we have used the same powerful and reproducible Real time PCR technique for fifteen years.

### EBV and RA immunosuppressive therapies

#### Anti TNF alpha

To test if TNF inhibitors impair EBV control, we have previously followed 128 RA patients under TNF inhibitors and observed stability of EBV load over time in most patients [11]. Therefore we did not expect to see any increase of B cell lymphoma incidence in RA patients under TNF inhibitors. This prediction is supported by many meta-analyses and registry surveys [32,33,38–40].

#### Abatacept

Abatacept down regulates T cell activation in RA, as cyclosporine does in transplants patients. Therefore, monitoring EBV load is very interesting in RA patients under abatacept.

In this study, we found no increased EBV load, as opposed to what has been detected with belatacept [22]. This is consistent with the fact that abatacept has a lower affinity than belatacept for CD80/86 [21].

Thus, our results predict no increase of lymphoma standardized incidence ratios (SIR) under abatacept therapy.

This finding is consistent with cumulative safety databases for abatacept intravenous administration in RA which show a number of lymphomas similar to that expected in the general US population [41].

In registries, only few studies describe lymphoma in patients treated by Abatacept [42]. In the meta-analysis of Smitten et al., patients with RA are at increased risk of lymphoma compared to a healthy population. However, the cumulative SIR for lymphoma is not compared with external RA cohorts limitating the significance of this study. SIR of lymphoma were similar with those observed in other registries suggesting that biologics are not associated with any further increase to the already elevated lymphoma occurrence in RA [33].

#### Tocilizumab

Our study showed significant (p = 0.021) decrease of EBV load under Tocilizumab, as published by others [23,24]. In a recent cross-sectional case-control study, Erre GL et al. studied EBV load by Real time PCR in one hundred thirty-five Sardinians (77 RA patients and 58 healthy donors, HDs). EBV DNA load was significantly higher in RA PBMCs than in HD PBMCs. Remarkably, PBMCs from RA patients under Tocilizumab, have significantly lower EBV viral loads than PBMCs from RA patients under other immunosuppressors (p = 0.03)[43].

The decrease in EBV load noted with tocilizumab might be due to a loss of memory B cells, although IL-6 signaling is probably not required for their maintenance. In this study, we did not measure the number of memory B cells among PBMNCs.

Our data does not predict increased incidence of EBV associated lymphoma in RA patients under Tocilizumab therapy. This seems to be in accordance with safety data: a meta-analysis did not show LPD enhancement under Tocilizumab, even after one year [38]. Only one case of EBV reactivation has been described in Japan under Tocilizumab therapy [44]. In the Japanese registries, lymphoma SIR did not significantly differ between the different biologics [45].

### EBV Load and DAS 28 activity

Our study finds no significant association (Abatacept p = 0.110, Tocilizumab p = 0.149,) between disease activity (as assessed by the DAS 28 index) and EBV load.

### Limitation of the study

The main limitation of our study is the important number of patients who were lost, most of them because of treatment lack of efficiency. Only 13/55 (23%) patients under abatacept completed the study. Ten of them were put in the tocilizumab arm. In the tocilizumab study, 8/35 (23%) patients completed the study. None of them developed lymphoma.

Larger scale and longer term studies are necessary to confirm the absence of EBV associated immunosuppression lymphoma under Abatacept and Tocilizumab.

## Conclusion

We monitored EBV load in PBMNCs of 55 RA patients under Abatacept and 35 patients under Tocilizumab for durations ranging from 6 months to 3 years. EBV load was stable over years under Abatacept and diminished significantly over time under Tocilizumab.

Therefore, these two medications are not expected to increase the incidence of immunosuppression lymphoma in RA patients.

## Supporting information

S1 Fig Association between DAS28 and EBV load in RA patients treated with Abatacept. No association could be demonstrated.
(TIFF)

S2 Fig Association between DAS28 and EBV load in RA patients treated with Tocilizumab. No association could be demonstrated.
(TIFF)

S1 Table EBV load in RA patients treated with Abatacept. Epstein Barr virus copy number per 500ng PBMC DNA was assayed in 55 patients under abatacept.
(XLSX)

S2 Table EBV load in RA patients treated with Tocilizumab. Epstein Barr virus copy number per 500ng PBMC DNA was assayed in 35 patients under tocilizumab.
(XLSX)
