# Supplementary figures and images for: Long term treatment with abatacept or tocilizumab does not increase Epstein-Barr virus load in patients with rheumatoid arthritis - A three years retrospective study
Source: PLoS One. 2017 Feb 15;12(2):e0171623. doi: 10.1371/journal.pone.0171623 (PMC5310777; doi:10.1371/journal.pone.0171623)

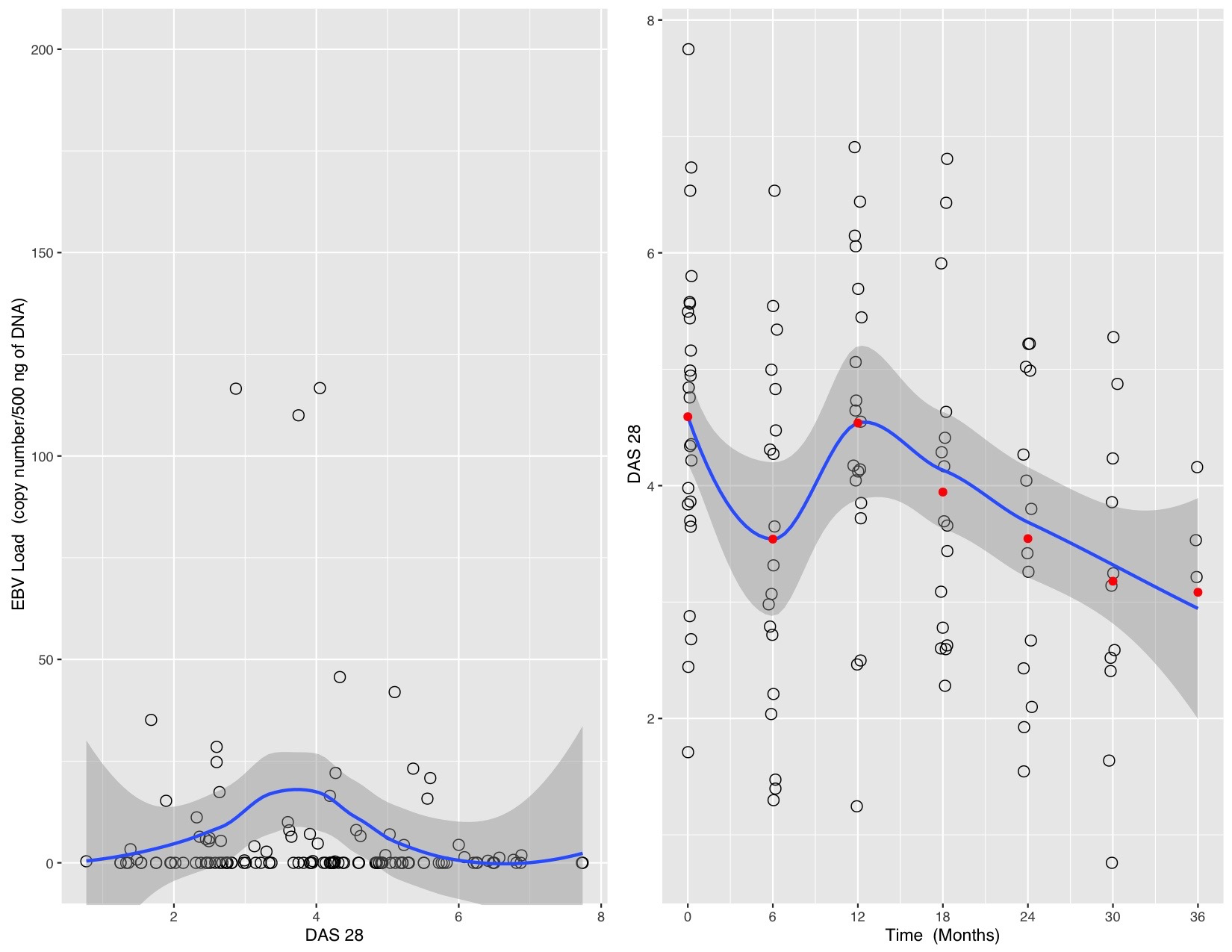

Supplement: S1 Fig — No association could be demonstrated. (TIFF) [file pone.0171623.s001.tiff]

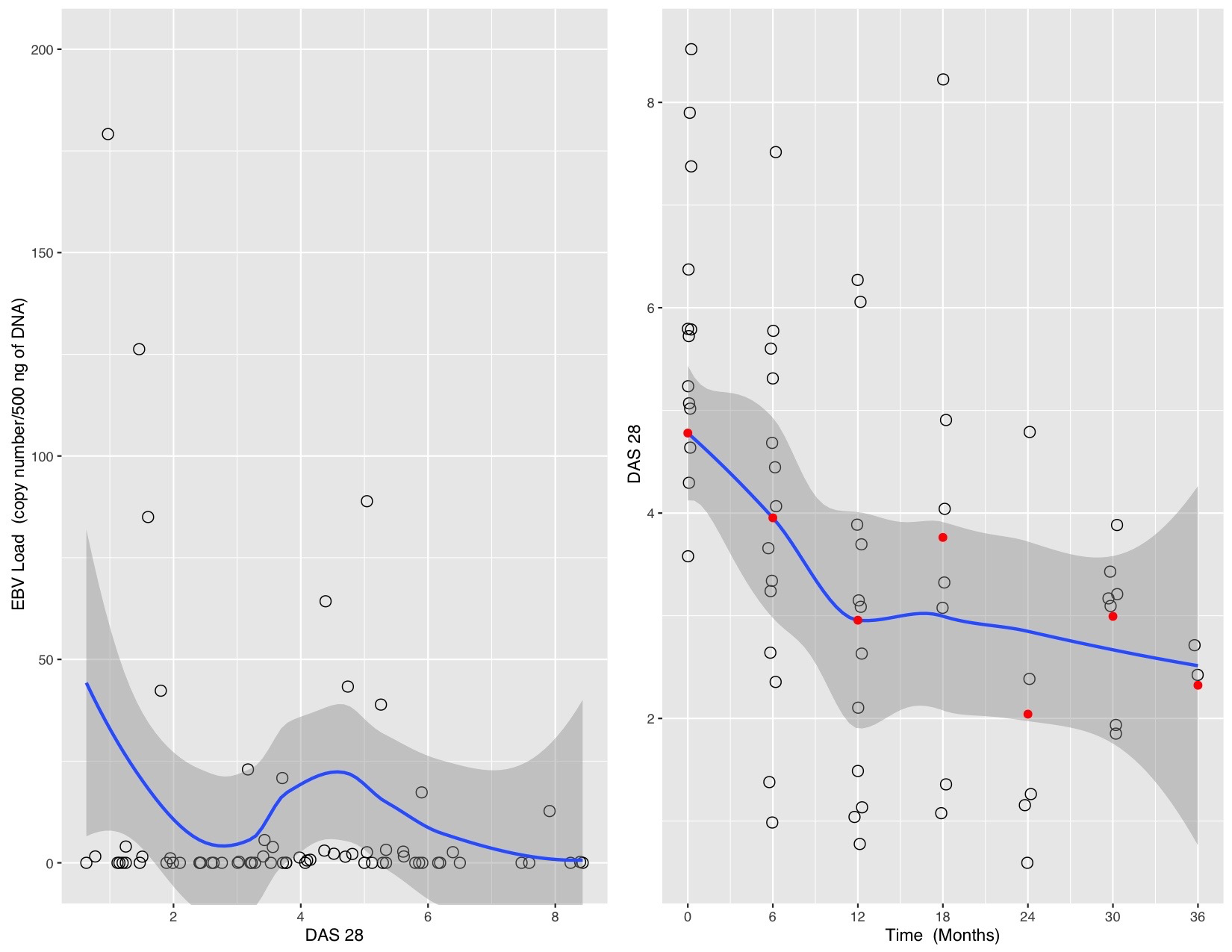

Supplement: S2 Fig — No association could be demonstrated. (TIFF) [file pone.0171623.s002.tiff]
